# Molecular mechanisms of flowering phenology in trees

**DOI:** 10.48130/FR-2023-0002

**Published:** 2023-01-16

**Authors:** Jun Wang, Jihua Ding

**Affiliations:** College of Horticulture and Forestry, Hubei Hongshan Laboratory, Hubei Engineering Technology Research Center for Forestry Information, Huazhong Agricultural University, Wuhan 430070, China

**Keywords:** Flowering phenology, Perennial tree, Juvenility, Reproductive competence, Seasonal growth

## Abstract

Flower initiation is a phenological developmental process strictly regulated in all flowering plants. Studies in *Arabidopsis thaliana*, a model plant organism in plant biology and genetics, and major cereal crops have provided fundamental knowledge and understanding of the underlying molecular mechanisms and regulation in annuals. However, this flowering process and underly molecular mechanisms in perennials are much more complicated than those in annuals and remain poorly understood and documented. In recent years, the increasing availability of perennial plant genomes and advances in biotechnology have allowed the identification and characterization of flowering-associated gene orthologs in perennials. In this review, we compared and summarized the recent progress in regulation of flowering time in perennial trees, with an emphasis on the perennial-specific regulatory mechanisms. Pleiotropic effects on tree growth habits such as juvenility, seasonal activity–dormancy growth, and the applications of tree flowering phenology are discussed.

## Introduction

Flowering time is a complicated, environmentally responsive trait, which can impact the fitness and survival of all flowering plants^[1]^. The timing of flowering is determined by endogenous genetic factors, as well as various environmental signals, such as photoperiod, temperature, and stress^[2]^. Studies in *Arabidopsis thaliana*, a model plant, have provided a basis for understanding plant flowering regulation in annual plants, in which flowering time is precisely controlled by a gene regulatory network comprising more than 300 genes^[3]^. These genes are involved in complex signal pathways including the autonomous, age, circadian clock, and gibberellin (GA) pathways that respond to intracellular and intercellular signals, and vernalization, ambient temperature, and photoperiod pathways that react to environmental cues. Moreover, with some notable exceptions, genes with analogous functions and similar molecular mechanisms found in *Arabidopsis* have conserved functions in flowering regulation in annual crop species. Recent reviews have provided detailed descriptions of flowering genes and mechanisms in annual plants^[4−8]^. These provide a basis for the understanding of gene networks controlling the flowering phenology of trees discussed in this review.

**Figure 1:**
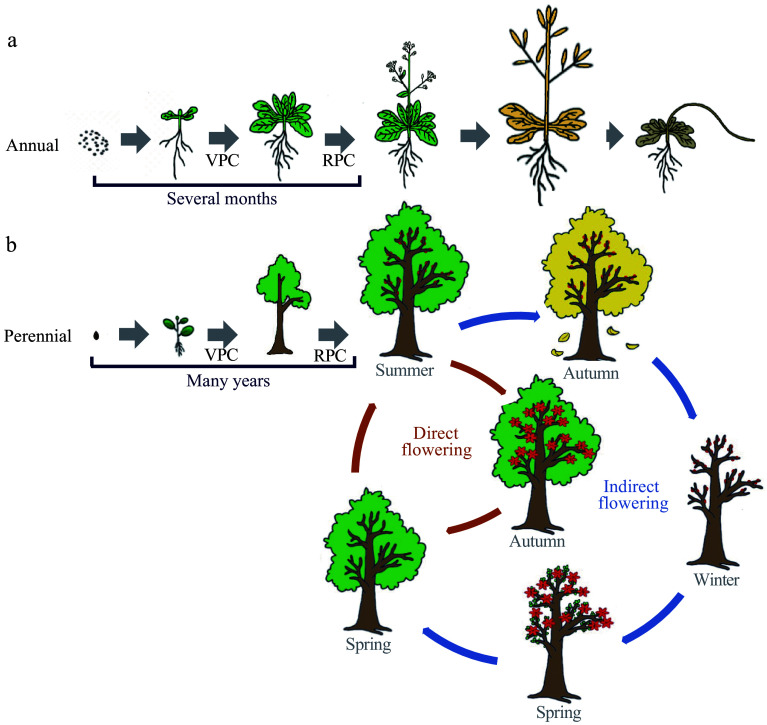
Comparison of flowering phenology between annual and perennial woody plants. The life cycle of flowering plants can be considered as a succession of distinct growth phases: vegetative growth, followed by a reproductive phase and eventually seed set and senescence. Annuals are fast cyclers and only need several months from the stage of vegetative development to flowering, and complete their life cycle within one growing season (a). While perennial woody plants experience a prolonged vegetative phase with many years until the first onset of flowering. Following first-time flowering, trees undergo seasonal flowering throughout their lifespan (b). Tree’s seasonal flowering can mainly be divided into 'direct' and 'indirect' flowering types, based on whether the development from initiation to emergence is interrupted or includes a period of rest. The 'indirect' flowering is common among temperate/boreal trees. It displays extended periods between flower initiation and flower blooming, in which flowers initiate in the summer are dormant through the winter, and the trees do not blossom until the following spring. In comparison, 'direct' flowering is common among subtropical or tropical evergreen species. They finish their complete reproductive cycles during a single growing season without dormancy or a rest period. VPC, vegetative phase change; RPC, reproductive phase change.

Most annual or biennial plants are monocarpic, flowering only once in their life cycle before death^[2]^ (Fig. 1). Unlike annual plants, woody perennial species are typically polycarpic and undergo repeated vegetative and reproductive growth cycles^[9]^. Perennial trees take several years to undergo the juvenile to adult phase change to acquire reproductive capability^[10]^. Following first-time flowering, trees flower annually throughout their lifespan (Fig. 1). Therefore, flowering is split into two dimensions in perennial trees: the first onset of flowering after many years of juvenility, and seasonal flowering after reproductive maturity (Fig. 1). The very long lifespan and polycarpic growth habits require a more complex regulatory network to synchronize environmental cues and mediate the appropriate flowering time^[11]^. The availability of increasing genome assemblies for trees now allows the identification of flowering phenology-associated gene orthologs in perennial plants. Many flowering genes have been functionally characterized in perennial trees, and conserved as well as functionally divergent genes involved in flowering have been found. This review summarizes the current understanding of flowering time regulation in perennial trees. Moreover, we discuss the pleiotropic effects on tree growth habit such as juvenility, seasonal activity–dormancy growth, and the applications of tree flowering phenology.

## The juvenile-to-adult phase transition in perennials and its correlation with reproductive competence

The life cycle of flowering plants can be considered a succession of distinct growth phases: vegetative growth, followed by a reproductive development and seed set, and eventually senescence (Fig. 1). The length of these phases varies among species and is particularly extended in perennial plants. Annuals progress quickly from vegetative to reproductive stage to complete their life cycle in one growing season. While perennial woody plants undergo a prolonged vegetative phase varying from a few years to several decades until the first onset of flowering^[10]^ (Fig. 1). The precise development phase transitions are essential for the success of plant adaptability, survival, and reproduction. Floral induction depends on the transition from the juvenile-to-adult vegetative phase (vegetative phase change, VPC), called the age pathway in flowering regulation^[12,13]^. In *Arabidopsis*, VPC and flowering transition are regulated by the sequential activity of two microRNAs, miR156 and miR172, and their respective target genes^[13,14]^. With the aging of the plant, a gradual decline in miR156 abundance occurs in accordance with a steady accumulation of *SQUAMOSA PROMOTER-BINDING PROTEIN* (*SBP*)*-LIKE* (*SPL*) transcription factors (TFs)^[13,14]^. miR156 reduction is also coupled with the gradual accumulation of miR172, which can repress *APETALA2* (*AP2*)*-like* TFs^[15,16]^. *SPL* and* AP2-like* gene expression is regulated by diverse flowering signals and their products form the molecular output of a pathway that regulates VPC and flowering initiation^[17,18]^. The miR156/miR172 module is conserved and regulates VPC in several other crop species^[19]^.

**Figure 2:**
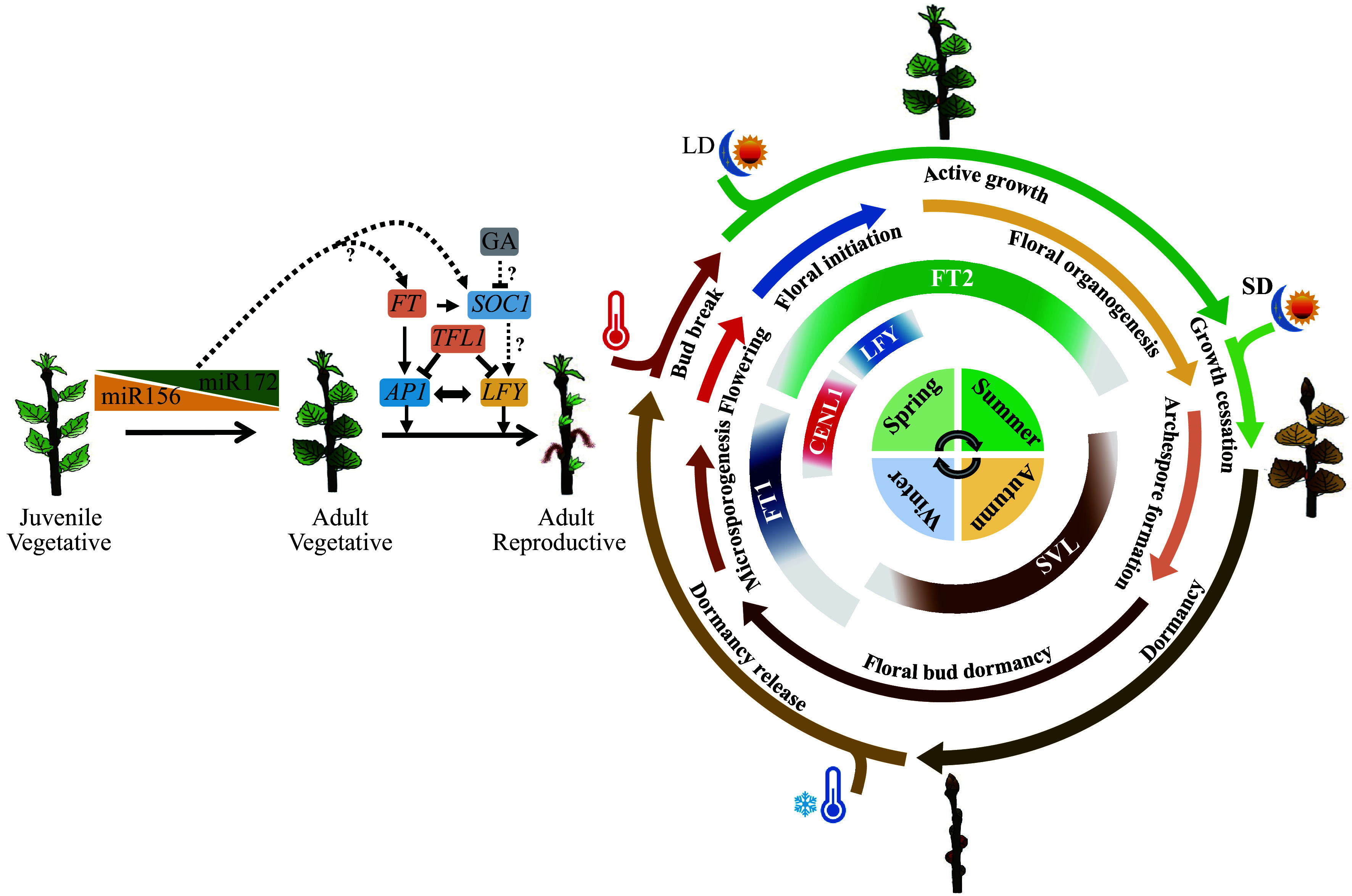
Molecular pathways of flowering phenology, and their shared mechanisms in seasonal vegetative growth regulation in *Populus*, the model tree for perennial plant phenology study. Flowering is split into two dimensions: one is the first onset of flowering after many years of juvenile and adult vegetative growth; another is seasonal flowering after reproductive maturity. Conserved to herbaceous plants, the juvenile to adult vegetative phase change is mainly regulated by two microRNAs, miR156 and miR172. The first onset of flowering is controlled by *FT/TFL1* family genes and their downstream integrators such as *AP1* and *LFY*. As trees will undergo a long period of adult vegetative stage until floral induction, how miR156/miR172 module regulate age-dependent flowering in trees remains an open question. Unlike in *Arabidopsis*, GA usually inhibits flowering in diverse woody angiosperms. Whether GA regulates flowering through *SOC1-like* genes, and do* SOC1-like* genes control reproductive competence in trees needs further investigation. For seasonal flowering, the expression of flowering integrator genes, such as* FT* (*FT1* and *FT2*), *CENL1, SVL*, and *LEAFY* (*LFY*), are controlled by seasonal cues like photoperiod and temperature. The specific expression patterns of these genes ensures the tree undergoes floral initiation at a specific time of the year. Meanwhile, these flowering integrator genes also play key roles in the seasonal activity-dormancy vegetative growth, including photoperiod-induced growth cessation of shoot apex at the end of summer, dormancy induction in autumn, cold-induced release of dormancy in winter, and warm temperature-induced bud burst in spring. Thus, trees have evolved an ability to incorporate the environmental signal to different developing events. the diagram sketch of seasonal growth from the inside out represent seasons, the expression pattens of flowering integrator genes, the seasonal flowering events, the seasonal vegetative growth events and environment signals such as photoperiod (LD and SD, long day and short day) and temperature (high and low) respectively.

Perennial woody plants experience a long period of vegetative growth before the first flower onset; thus, it is of more pragmatic value to study phase transitions of perennial woody plants. Studies of broad-leaved trees, such as *Populus canadensis, Acacia confusa*, *A. colei*, *Hedera helix*, *Eucalyptus globulus*, *Quercus acutissima*, *Folium mori, Mangifera indica*, *Malus hupehensis*, *Persea americana*, and *Macadamia integrifolia*, have shown that miR156 and miR172 have similar expression trends with age^[20−23]^, suggesting that miR156 and miR172 are common to almost all major plant taxa and their roles in the control of VPC appears conserved (Fig. 2). miR156 overexpression in both *P. canadensis* and* P. tremula* × *alba* prolongs the juvenile phase, providing a genetic support for its role in VPC in trees^[20,24,25]^. However, recent studies in a gymnosperm *Pinus tabulaeformis* showed that the expression pattern of miR156 and its target genes showed no correlation with age, suggesting diversity of VPC control in gymnosperm trees^[26]^. In *Arabidopsis*, the miR156/miR172 module showed strong connections between VPC and reproductive competence^[12,13]^. However, the relationship between VPC and floral induction in perennial plants is unclear. Morphological changes during VPC have been comprehensively characterized in* P. tremula × alba* using miR156 overexpression and knockdown transgenic plants, and the onset of adult traits already begins within three months of growth^[25]^. This phenomenon is interesting as it corrected our traditional understanding that trees have a long juvenile stage. If VPC is completed at the early stage, trees undergo a long period of the adult vegetative stage until floral induction. This raises a question on how the miR156/miR172 module coordinates these two processes. Although ectopic expression of *SPL* gene from* Citrus clementina* and* Eriobotrya japonica* could promote flowering in *Arabidopsis*^[27,28]^. Thus far, however, reports confirming that the miR156/miR172 module and related genes regulate floral initiation in trees are rare. To what extent miR156 and miR172 and their targets function in the first flowering of trees remains to be clarified (Fig. 2). Besides the age pathway, *AP2-like* genes contribute to polycarpy in *Arabis alpina,* which provides a valuable clue in understanding the molecular basis of the polycarpic growth habit of woody trees^[29]^.

## Advances in molecular mechanisms of reproductive competence in trees

Most knowledge about the molecular mechanisms of flowering time comes from studies in the annual plant *Arabidopsis*, in which flowering initiation is induced by multiple pathways that converge to a few integrator genes, such as *FLOWERING LOCUS T* (*FT*) and *SUPPRESSOR OF OVEREXPRESSION OF CONSTANS 1* (*SOC1*). These genes act as floral pathway integrators to activate downstream floral meristem identity genes, such as *LEAFY* (*LFY*) and* APETALA1* (*AP1*), and cooperate to promote flowering^[6,30]^. Due to a lack of availability of molecular resources, the molecular mechanisms that regulate reproductive competence have not been widely explored in perennials. The most common way is to study the functional conservation of *Arabidopsis* genes regulating flowering by ectopic expression of these genes in transgenic trees (Table 1). The gene* FT* and its family member *TERMINAL FLOWER 1* (*TFL1*) have contributed largely to our understanding of the molecular mechanisms that regulate reproductive competence in perennials. *FT* and *TFL1* encode a pair of flowering regulators of the phosphatidylethanolamine-binding protein family^[31]^.* FT* promotes the reproductive transition and flowering, whereas* TFL1* represses flowering^[32]^. The antagonistic roles of *FT*/*TFL* in mediating flowering signals have been documented in all angiosperm species examined^[5,33]^. In poplar trees, leaves in adult shoots have higher expression levels of *FT* than leaves in juvenile shoots^[34]^. Overexpression of *FT* orthologs can induce premature flowering in many perennial species^[34−44]^ (Table 1). In contrast, transgenic *M. domestica*, *P. trichocarpa*, and *Actinidia chinensis* plants with reduced *TFL1* expression accelerated flowering and shortened the length of vegetative growth before first flowering^[45−47]^ (Table 1). Such an antagonistic function of *FT* and *TFL1* observed in trees suggests their functional conservation in reproductive competence in perennial species.

**Table 1:** Functional orthologs of flowering integrator genes identified in perennial trees.

Species	Gene	Construction	Flowering	Other effects	References
Apple (*Malus pumila* Mill.)	*MdFT1*	Overexpression	^A,N^Induction		[43]
	*MdTFL1, MdTFL1.1* *MdCENa, MdCENb*	CRISPR/RNAi	^A,T,N^Repression		[45,159−161]
	*AFL1, AFL2*	Overexpression	^A,N^Induction		[162,163]
	*MdDAMa, MdDAMb, MdDAMc* *MdSVPa, MdSVPb*	RNAi	^N^Induction	Regulates bud dormancy	[135]
	*MdFLC1* *MdFLC3*	Overexpression	^A^Repression	Juvenility regulation	[164]
Avocado (*Persea americana*)	*PaFT*	Overexpression	^A^Induction		[165]
Blueberry (*Vaccinium corymbosum* L.)	*VcFT*	Overexpression	^T,N^Induction		[166]
Birch *(Betula*)	*BpAP1*	Overexpression	^N^Induction		[68]
Citrus (*Citrus sinensis*)	*CsTFL*	Overexpression	^A^Repression		[167]
Citrus (*Citrus clementina*)	*CsAP1*	Stress-inducible promoter	^N^Induction		[168]
	*CsLFY*	Stress-inducible promoter	^N^Induction		[168]
	*CsSL1, CsSL2*	Overexpression	^A^Induction		[52]
Dogwood (*Cornus* L.)	*CorcanTFL1, CorfloTFL1*	Overexpression	^A^Repression		[169]
Eucalyptus (*Eucalyptus* spp.)	*AtFT*	Overexpression	^N^Induction		[152]
	*PtFT1*	Overexpression	^N^Induction		
	*ELFY*	CRISPR		Affects floral development	[170]
	*EgSVP*	Overexpression	^A^Repression	Affects floral development	[171]
Fig (*Ficus carica*)	*FcFT1*	Overexpression	^T^Induction		[172]
Magnoliaceae	*MawuAP1*	Overexpression	^A^Induction		[173]
Grapevine (Vitis spp.)	*VvTFL1A*	Overexpression	^A^Repression		[174]
	*VvFT*	Overexpression	^A^Induction		
Japanese apricot (Prunus mume)	*PmFT*	Overexpression	^A^Induction		[175]
	*PmTFL1*	Overexpression	^A^Repression		
Jatropha (*Jatropha curcas* L.)	*JcFT*	Overexpression /RNAi	^A,N^Induction		[36,176]
	*JcLFY*	Overexpression	^A,N^Induction	Affects floral fruit and seed development	[177,178]
	*JcAP1*	Overexpression	^A^Induction		[179]
	*JcTFL1a, JcTFL1b, JcTFL1c*	Overexpression	^A,N^Repression		[180]
	*JcTFL1*	RNAi	^N^Induction		
Kiwifruit (*Actinidia* spp.)	*AcFT1, AcFT2*	Overexpression	^A,N^Induction		[181,182]
	*AcCEN1, AcCEN2,* *AcCEN3, AcCEN4*	Overexpression/CRISPR	^A^Repression		[47,181,182]
	*AcBFT1, AcBFT2, AcBFT3*	Overexpression/CRISPR	^A^Repression	Affects dormancy and bud break	[182,183]
	*SVP1-4*	Overexpression	^A,T,N^Normal	Affects dormancy	[142,184,185]
	*AcSOC1e, AcSOC1f, AcSOC1i*	Overexpression	^A^Induction ^N^Normal	Affects dormancy	[54]
	*AcFLCL*	Overexpression/CRISPR		Regulate bud break	[123]
Litchi (*Litchi chinensis* Sonn.)	*LcFT1, LcFT2*	Overexpression	^A,T^Induction		[186]
London plane (*Platanus acerifolia*)	*PaFT*	Overexpression	^A^Induction ^T^Induction		[187]
Longan (*Dimocarpus longan* L.)	*DlFT1*	Overexpression	^A^Induction		[188]
	*DlFT2*	Overexpression	^A^Repression		
Loquat (*Eriobotrya japonica*)	*EjTFL1-1, EjTFL1-2*	Overexpression	^A^Repression		[189]
	*EjSOC1-1, EjSOC1-2*	Overexpression	^A^center		[51]
	*EjLFY-1*	Overexpression	^S^Induction		[190]
Mango (*Mangiferaindica* L.)	*MiFT1*	Overexpression	^A^Induction		[191]
	*MiFT2; MiTFL1-1, MiTFL1-2,* *MiTFL1-3, MiTFL1-4*	Overexpression	^A^Repression		[191,192]
Norway spruce (*Picea abies*)	*PaFTL1, PaFTL2*	Overexpression	^A^Repression		[193]
	*PaFTL2*	Overexpression		Control growth arrest	[119]
Olive (*Olea europaea* L.)	*OeFT1, OeFT2*	Overexpression	^A^Induction		[194]
Peach (*Prunus persica* L.)	*PpTFL1*	Overexpression	^A^Repression		[195]
	*PpAP1*	Overexpression	^A^Induction		[196]
	*PpFT*	Overexpression	^A^Induction		[197]
Pear (*Pyrus communis *L.)	*PcTFL1-1, PcTFL1-2*	RNAi	^N^Induction		[151]
	*PcFT2*	Overexpression	^T^Induction ^N^Normal	Regulate vegetative growth	[198]
	*PcTFL1.1*	CRISPR	^N^Induction		[159]
Pomegranate (*Punica granatum* L.)	*PgTFL1, PgCENa*	Overexpression	^A^Repression		[199]
Poplar (*Populus* spp.)	*FT1, FT2*	Overexpression/CRISPR	^N^Induction	FT1 regulates bud break; FT2 regulates growth cessation	[34,44, 103,117]
	*LAP1*	Overexpression/RNAi	^A^Induction	Regulates growth cessation	[200]
	*PopCEN1, PopCEN2*	Overexpression/RNAi	^N^Repression	Regulates bud break	[46]
	*SVL*	Overexpression/RNAi	^N^Repression	Regulate growth cessation, dormancy and bud break	[139,140, 143,144]
Rubber trees (*Hevea brasiliensis*)	*HbMFT1*	Overexpression	^A^Repression		[201]
Sweet Cherry (*Prunus avium* L.)	*PavFT*	Overexpression	^A^Induction		[202]
	*PavSVP*	Overexpression	^A^Repression		[203]
	*PavSOC1*	Overexpression	^A^Induction		[55]
	*PaAP1*	Overexpression	^A^Induction		[204]
Tea-oil tree (*Camellia oleifera* Abel.)	*CoFT1*	Overexpression	^A^Induction		[205]
Trifoliate orange (*Poncirus trifoliate*)	*CiFT*	Overexpression	^A,N^Induction		[35,38,206]
N, A, T and S represent function assessed in native plant, Arabidopsis, tobacco and strawberry respectively.					

*SOC1* is another floral pathway integrator that integrates multiple flowering signals, including age-dependent signals in which* SPL9* and miR156 are involved^[48]^. Thus, it is reasonable to speculate that *SOC1-like* genes would have roles in the cooperation of VPC and reproductive transition in trees. Recent studies in the perennial conifer *P. tabulaeformis* have identified 33 age-dependent TFs, among which 11 belong to the MADS-box family including* SOC1-like* genes^[49]^. Combined with transcriptome association analysis and genetic confirmation, the *SOC1-like* gene* MADS11* was confirmed as a regulatory mediator of VPC in pine^[49]^. Thus far,* SOC1-like* genes have been widely studied in many angiosperm perennial trees. Many *SOC1-like* genes from different tree species have been confirmed to complement the late flowering of the *soc1* mutant in *Arabidopsis*, suggesting their conserved roles in flowering induction^[50−54]^. However, their roles in reproductive competence in native plants are less known. Ectopic expression of *AcSOC1* in *Actinidia chinensis* failed to induce precocious flowering^[54]^. Instead, *SOC1-like* genes were associated with bud dormancy maintenace and dormancy released in many trees^[54−56]^, suggesting their functional diversification in woody plants. Whether and how *SOC1-like* genes regulate flowering induction in trees requires further investigation (Fig. 2).

Besides* FT* and *TFL1*, overexpression of downstream floral meristem identity genes can overcome several years of the juvenile period in multiple woody species. For example, *LFY* plays an important role in both flowering initiation and floral meristem differentiation^[57,58]^. *LFY* homologs have been studied in many perennial trees, and their gene overexpression causes early flowering in hybrid aspen, citrus, litchi, and so on^[59−64]^. *AP1* is both a floral meristem identity and a floral organ morphology gene, and possibly regulates flowering^[65−67]^. In perennial trees, though not all, overexpression of *AP1* homologs can also induce flowering^[61,68]^. Therefore, orthologs of these floral initiation genes have conserved functions in regulating the first flowering after a long period of juvenility (Fig. 2).

The phytohormone GA plays a major role in flowering regulation in *Arabidopsis*. It promotes flowering by inducing *SOC1* and *LFY* expression under short-day conditions^[69,70]^. Increased LFY activity causes reduced GA levels by directly up-regulating the GA catabolic enzyme *EUI-LIKE P450 A1* (*ELA1*) as well as *GA2 oxidases*, which in turn enables accumulation of DELLA proteins that complex with the SBP transcription factor SPL9 to activate *AP1*^[71]^*.* Thus, the GA plays dual opposite roles on flower formation onset in *Arabidopsis*. It promotes termination of vegetative development while inhibiting flower formation^[71]^. The role of GA in the floral initiation of woody perennials seems more complicated. It often inhibits flowering in diverse woody trees^[72,73]^. As perennial trees have two dimensions of flowering initiation: the first onset of flowering after many years of juvenility, and the seasonal flowering once reproductive maturity is reached. Most reports that showed GA inhibits floral induction refer to the seasonal flowering onset. This is also supported by the results of GA treatment of different physiological age of trees. Application of GA inhibitor only induce flowering in mature shoots in *Eucalyptus nitens* and *Populus deltoides,* but it appears to be inefficient on juvenile shoots^[74,75]^. In this case, the negative role of GA on seasonal flowering switch in woody angiosperms may be similar to the negative function of GA on flower formation in *Arabidopsis*. However, unlike its positive roles on vegetative termination in* Arabidopsis*, GA likely also inhibits the first onset of flowering in many woody angiosperms. Application of exogenous GAs in several perennial species can even cause a reverse from reproductive to vegetative development^[76]^. Previous studies have shown that juvenile shoot apices contained higher levels of endogenous GAs than adult shoot apices^[76]^. In grapevine, the GA inhibition of tree flowering is confirmed by an early flowering grapevine mutant that is defective in a grapevine homolog of the *Arabidopsis* gene* GA INSENSITIVE* (*GAI*), a key gene involved in GA signal transduction^[77]^. However, similar to *Arabidopsis*, GA appears as a flowering activator in conifers, and it is widely applied to stimulate flowering for breeding purposes^[78]^. Overall, the molecular mechanism of GA signals in the reproductive competence of trees is still an unsolved mystery (Fig. 2).

## Diversity of seasonal flowering phenology and their environmental drivers in trees

After perennials become capable of reproduction, they periodically flower with seasonal changes. Trees have evolved to time their flowering in appropriate seasons to adapt to geographically different environments. Thus, there is a rich diversity of flowering phenology from temperate to tropical climates^[79]^. In temperate regions, trees synchronize their flowering time to coincide with appropriate seasons by responding to seasonal environmental cues, particularly temperature and photoperiod, and flowering usually peaks from spring to early summer^[80]^. In tropical and subtropical forests where seasonal environmental cues are less available, there is a wide variation in flowering time patterns and its climatic drivers. Flowering can be seasonal or aseasonal with variation across years. For example, in tropical deciduous forests in India, five flowering types occur and the variation in flowering is relative to leaf flushing^[81]^. In seasonally dry tropical forests, flowering is driven by water availability, and flowering peaks usually occur at the end of the dry season or the beginning of the wet season^[82,83]^. Sometimes, the effect of climate on flowering phenology in subtropical forests is difficult to predict because of wide variations in rainfall seasonality. For instance, in Australia, flowering phenology varies among species, with both seasonally dynamic and spatially variable, driven by temperature, rainfall, and soil/substrate moisture^[84]^. Although there is no dry season in the Atlantic rainforest in Brazil, there is still clear seasonality in leafing and reproductive events that might be affected by slight changes in photoperiod and/or temperature^[85]^. Mass flowering occurs in some aseasonal Asian and South American tropical forests^[86,87]^. The differences in flowering phenological patterns observed among temperate forests, tropical dry forests, and tropical rainforests highlight differences in plant response to environmental cues. These environmental cues not only include the relatively stable seasonal environmental signals, such as temperature and photoperiod, but also biotic and abiotic stresses, such as drought, heat, and salinity. Such varied flowering phenology and their environmental drivers in trees support the idea that the altering flowering time is an evolutionary strategy for plant to maximize the chances of reproduction under diverse stress conditions^[2]^. We should keep in mind that the above observations of flowering phenology focus on flowering time rather than the time of floral initiation. Although flowering time is closely related to flower initiation, the environmental drivers can differ.

## Molecular basis of seasonal flowering phenology in trees

Flowering has been studied most extensively in *Arabidopsis*, in which temperature and photoperiod are two major environmental signals that regulate flowering initiation. However, knowledge of seasonal flowering initiation in perennial trees is scarce. On one hand, annual and perennial plants have different growth habits: most annual or biennial plants are monocarpic, whereas perennial species are typically polycarpic. These different growth habits are reflected by flowering patterns^[88]^. All meristems of annual plants transform to floral meristems, and the life cycle is completed within one year (Fig. 1). Perennial trees have asynchronous differentiation behavior of meristems, with some committing to reproductive development, whereas others retain vegetative growth^[9,89]^. Seasonal tree flowering can be mainly divided into 'direct' and 'indirect' flowering types, based on whether the development from initiation to emergence is interrupted or includes a period of rest^[88]^. 'Indirect' flowering is common among temperate trees (poplar, cherries, pears, plums, apples, etc.)^[90,91]^. It displays extended periods between flower initiation and blooming, in which flowering is initiated in summer, trees are dormant by winter, and trees do not blossom until the following spring (Fig. 1). In comparison, 'direct' flowering species (mango, jujuba, etc.) complete their reproductive cycles in a single growing season without dormancy or a rest period^[92,93]^ (Fig. 1). On the other hand, seasonal flowering phenology is not mutually independent from other phenological events, such as seasonal growth cessation, dormancy, leaf flushing, and fruiting. Such a long flowering time accompanied by a complex natural environment and various phenological events, as well as asynchronous development of the axillary meristem, makes it more challenging to determine the time of flowering initiation and their environmental drivers.

Although orthologs of floral pathway integrator genes, such as *FT*, *LFY*, and* AP1*, have been isolated from many trees, and their functions on reproductive competence are conserved among species (discussed in an earlier section of this review), much less is known about the molecular regulation of seasonal flowering. Applying advances in transcriptomics is an effective strategy to reveal the underlying mechanism of dynamic environmental responses in plants. Recently, a molecular phenology approach that monitors seasonal gene expression patterns in nature has been increasingly applied in a range of plants to explore plant responses to fluctuating natural environments^[79,94]^. These field-based seasonal transcriptomes provide ideal maps for associating genes with flowering phenology^[95−101]^. This approach was also used to successfully identify environmental signals driving flowering in different tree species. For example, seasonal transcriptome studies in *Fagus crenata* showed that the expression levels of *FT*, *LFY*, and* AP1* orthologs display clear between-year fluctuations^[100]^. These between-year fluctuations in gene expression coincided with the nitrogen change of current-year shoots. Plants fertilized with nitrogen can induce the expression of these three genes in *F. crenata* and flowered in two consecutive years. This result suggests that nitrogen is a key regulator of flowering initiation in this species^[100]^. Similarly, we recently characterized the annual transcriptome dynamics of the subtropical hardwood tree *E. dunnii* in natural field environments. Our transcriptome analysis, combined with geographical distribution, environmental cues, and heterologous transformation analyses, suggests that low temperature is one of the environmental triggers for its seasonal flowering^[102]^. Things are usually more complex because of gene duplication and sub-functionalization. For example, in *Populus*, two *FT-like* genes have been identified: *PtFT1* and *PtFT2*. They have distinct seasonal expression patterns: *PtFT2* peaks in late spring until early summer, and its expression is regulated by photoperiod, whereas *PtFT1* is only induced by cold and peaks in late winter^[103]^. Both *PtFT1* and *PtFT2* can induce early flowering, suggesting their protein conservation in promoting flower initiation in *Populus*^[34,44]^. The expression pattern of *PtFT2* is more similar to *Arabidopsis FT*, both of which are regulated by photoperiod^[44]^. However, *PtFT1* is supposed to be a potential seasonal floral activator based on its seasonal expression pattern (Fig.2)^[32,103]^; however, more genetic and molecular evidence is needed to prove which one determines seasonal flowering initiation. In the future, such a molecular phenology strategy, combined with approaches in molecular biology, ecology, and mathematical modeling, will be useful to dissect the environmental factors regulating flowering traits in different climate zones.

## Seasonal activity – dormancy growth and flowering share mechanisms

Trees native to temperate and boreal regions have evolved an important adaptive trait in which they undergo a seasonal activity–dormancy growth cycle for survival and growth^[104]^. This activity–dormancy cycle includes cessation of apical growth, bud set, and dormancy induction in the fall; maintenance and release of dormancy in winter; and bud burst in spring (Fig. 2). Different developmental phases in the cycle have different responses to multiple environmental factors, adaptations that enable synchronization of these phases to the local climatic conditions. Temperature and photoperiod are two primary environmental cues, with the contribution of these cues varying among species. In the last two decades, the molecular mechanism of seasonal activity–dormancy growth has been intensively studied in trees, especially in the model species *Populus*^[44,105−111]^. In *Populus*, the timing of growth cessation in the fall is primarily governed by photoperiod. The reason for this molecular mechanism breakthrough discovery is that *PtFT2*, besides promoting floral initiation, plays a key role in suppressing short-day induced growth cessation in the fall^[44]^. Since then, more genetic and molecular approaches have revealed a remarkable conservation of the photoperiod pathways in regulating growth cessation in *Populus* and controlling flowering time in *Arabidopsis*, originating from light perception by phytochromes (phyA and phyB), together with internal circadian clock genes such as *LATE ELONGATED HYPOCOTYL*, *GIGANTEA*, and *CONSTANS*, allowing plants to measure day length. Long photoperiods induce *PtFT2* expression in the leaves. PtFT2 can move from the leaves to the shoot apex through the phloem where it interacts with TF FDL1 to induce the expression of *LIKE APETALA1* and *AINTEGUMENTA-LIKE1*, which in turn activates the cell cycle genes and thus growth^[108,112−116]^. After growth cessation, the continuation of short days induces bud dormancy. The dormant buds need a certain period of cold temperature to release dormancy for bud burst in spring. Recent studies have shown that another paralog of* FT*, *PtFT1*, plays a key role in cold-induced dormancy release. Plants with knockout *PtFT1* showed inhibited dormancy release and delayed bud burst^[117]^ (Fig. 2).

Similar to the *FT/TFL1* function in flowering regulation, RNAi downregulation of the poplar *TFL1* homolog *CENTRORADIALIS1* (*CEN1*) or *CEN2* not only accelerated the first onset of flowering and increased the proportion of short shoots but also promoted dormancy release and advanced bud burst^[46]^. The antagonistically functioning paralogs *FT* and* TFL1* likely arose after duplication in the angiosperm lineage, and the flowering-promoting function of *FT* evolved after the divergence of angiosperms from gymnosperms 300 million years ago^[118]^. Notably, an *FT/TFL*-based mechanism for seasonal growth has also been observed in conifers such as spruce^[119,120]^. Two *FT/TFL1-like* genes (*PaFTL1* and *PaFTL2*) were identified in the conifer Norway spruce. Gene expression and population genetic studies have suggested that *PaFTL1* and *PaFTL2* act in concert to control perennial growth in Norway spruce. *PaFTL1* expresses in the meristem and prevents meristematic cell proliferation during active extension growth in summer, whereas *PaFTL2* attenuates extension growth in the fall^[119,121]^. Therefore, besides flowering time, *FT/TFL1* genes have evolved roles in controlling seasonal growth before angiosperms and gymnosperms diverged.

Many of the *Arabidopsis* MIKC MADS-box TFs are key regulators of reproductive development, including flowering time control, flower development, and inflorescence architecture. In* Arabidopsis*, the winter cold temperature response has a dominant effect on flowering time. Plants undergo vernalization to overcome prolonged cold, which suppresses flower initiation until cold acclimation is fulfilled by winter temperature under the control of a MADS-box protein, FLOWERING LOCUS C (FLC)^[30,122]^. Genes with sequence homology to *FLC* have been identified in many trees^[123−127]^. However, *FLC* appeared specific to the Brassicaceae lineage as no sequences were similar enough to be regarded as individual orthologs to the *FLC* gene in *Arabidopsis*. Whether and how genes in the autonomous and vernalization pathways have evolved roles in flowering regulation in many other species is still less known. It has been reported that the citrus* FLC-like* genes* CcMADS19* acts to regulate flowering by repressing the citrus *FT*^[128]^. Winter temperature response also exists in perennial trees from boreal and temperate climates, where plants undergo dormancy to overcome the harsh winter. Recent studies showed that *AcFLCL*, a kiwifruit *FLC-like* gene, is induced by cold and correlated with epigenitc changes to control budbreak in kiwifruit^[123]^. Besides *FLC*, another close homolog MADS-box TF, termed *dormancy-associated MADS-box* (*DAM*) plays a key role in this process. The *DAM* genes were discovered early in the nondormant *evergrowing* mutant of peach, which is incapable of going into dormancy^[129]^. *DAM* genes are orthologous to the floral repressor *SHORT VEGETATIVE PHASE* (*SVP*) of *Arabidopsis,* which is another MADS-box gene that plays a key role as a flowering repressor responding to ambient temperature^[130]^.* DAM*- and *SVP*-*like* genes have been characterized in many perennial species^[131−141]^, and the functions of these proteins in dormancy have been verified in transgenic plants of apple^[131,132,137,138]^, poplar^[139−140]^, and kiwifruit^[142]^. For example, a *Populus SVP* ortholog termed *SHORT VEGETATIVE PHASE-LIKE* (*SVL*) has been reported to play extensive roles in seasonal growth. It is not only involved in photoperiod-regulated growth cessation and bud set, but also plays crucial roles in seasonal dormancy initiation and release^[139,140,143]^. Besides dormancy regulation, recent studies have shown that *SVL* overexpression poplars delay the onset of flowering by several years in field-grown conditions^[144]^. However, *MdSVP* overexpression in apple delayed bud burst in spring, but flower development and time to first flowering were normal^[132]^. In *Arabidopsis*, *FT* also promotes flowering by another MADS-box gene, *SOC1*, the first gene to be activated in the shoot apex^[145]^. Similar to *SVP-like* genes, *SOC1*-*like* genes have also been associated with seasonal dormancy in perennial trees^[54−56]^. *SOC1* controls seasonal vegetative and reproductive growth in strawberry^[146]^. In poplar, one *SOC1* homolog *PTM5* is implicated in seasonality and spring wood formation, and another *SOC1* related gene promotes bud break^[56, 147]^. In apricot (*Prunus armeniaca*), a *SOC1*-*like* gene has been associated with chilling requirements during bud dormancy^[148]^. Functional studies of kiwifruit *SOC1*-*like* genes indicate that they affect the duration of dormancy but may not play a role in the transition to flowering^[54]^.

In brief, many of the genes involved in seasonal flowering are genes controlling seasonal activity–dormancy growth in trees. Thus, seasonal activity–dormancy vegetative growth and flowering may share a common mechanism in trees, although how these genes control tree flowering remains to be determined (Fig. 2).

## Future perspectives

Two decades ago, five central questions were raised about floral initiation in perennial trees^[91]^. To date, these questions have only been partially answered. However, some important underlying molecular mechanisms need to be further explored and understood. For example, 1) how flowering genes temporally and spatially regulate the first reproductive competency and seasonal initiation of flowering 2) How trees coordinate seasonal vegetative and reproduction growth, and to what extent do they share genetic pathways? 3) How trees adapt to local environments to precisely control flowering time in a season. The study on the molecular mechanisms of flowering phenology has been restricted due to a lack of genetic materials (e.g., a genome-wide mutant library), which take a long time to generate. Recent advances and applications of CRISPR/Cas9 technology have enabled the generation of knockout mutants of flowering-related genes^[149]^. Analysis of loss-of-function transgenics coupled with temporal–spatial expression analysis can provide important information to elucidate the functional roles of these genes during tree flowering. From the perspective of breeding, the prolonged juvenility of trees has greatly limited tree domestication. Thus far, many floral regulators can be used to regulate the switch from vegetative to reproductive growth in many tree species, such as *FT*,* TFL1*, *LFY*, and some MADS-box genes (Table 1). Gene manipulation has been used as a tool for accelerated breeding^[40,47,150−153]^. In addition to regulating flowering time, flowering time genes also have pleiotropic effects on plant growth and development, including seasonal growth, pollen fertility, and wood development. Understanding the molecular mechanisms of how flowering genes finely control these traits can help optimize the breeding strategies and processes.

With the progress of global warming, understanding how such a climate change impacts on the life cycle of organisms are critical for evaluating ecosystem vulnerability as the phenological shifts occur in the key life cycle of organisms^[154,155]^. Recently, the knowledge of the molecular basis of flowering genes in temperature response in *Arabidopsis. halleri* has been incorporated into a predictive model, which can be used to forecast flowering phenology under climate change^[156]^. With the increased knowledge of genetic architecture of flowering phenology in crops^[8]^, this approach can be used to predict the adaptation of crops to the changing environment. The flowering phenology of perennial trees has become a major contributor to climate change metrics applied to understand the impact of global climate changes on plant ecosystems^[154,155,157]^. However, what we understand of the genetic base of flowering phenology is biased toward temperate regions^[90,158]^. Improved mechanistic understanding of environmental drivers of plant flowering phenology in other ecosystems is urgently needed^[79]^. Integration of molecular knowledge of flowering phenology, climate data, and ecological perspectives can help us to assess the vulnerability of the ecosystem and predict risks of climate change.
